# Neutrophil contribution to spinal cord injury and repair

**DOI:** 10.1186/s12974-014-0150-2

**Published:** 2014-08-28

**Authors:** Virginie Neirinckx, Cécile Coste, Rachelle Franzen, André Gothot, Bernard Rogister, Sabine Wislet

**Affiliations:** GIGA Research Center, Neurosciences Unit, Nervous system diseases and treatment, University of Liège, Avenue de l’Hôpital, 1, 4000 Liège, Belgium; GIGA Research Center, Cardiovascular sciences Unit, University of Liège, Avenue de l’Hôpital, 1, 4000 Liège, Belgium; Hematobiology Department, University Hospital Liège, Avenue de l’Hôpital, 1, 4000 Liège, Belgium; GIGA Research Center, Stem Cells and Regenerative Medicine Unit, University of Liège, Avenue de l’Hôpital, 1, 4000 Liège, Belgium; Neurology Department, University Hospital Liège, Avenue de l’Hôpital, 1, 4000 Liège, Belgium

**Keywords:** Inflammation, Spinal cord injury, Neutrophils, G-CSF

## Abstract

Spinal cord injuries remain a critical issue in experimental and clinical research nowadays, and it is now well accepted that the immune response and subsequent inflammatory reactions are of significant importance in regulating the damage/repair balance after injury. The role of macrophages in such nervous system lesions now becomes clearer and their contribution in the wound healing process has been largely described in the last few years. Conversely, the contribution of neutrophils has traditionally been considered as detrimental and unfavorable to proper tissue regeneration, even if there are very few studies available on their precise impact in spinal cord lesions. Indeed, recent data show that neutrophils are required for promoting functional recovery after spinal cord trauma. In this review, we gathered recent evidence concerning the role of neutrophils in spinal cord injuries but also in some other neurological diseases, highlighting the need for further understanding the different mechanisms involved in spinal cord injury and repair.

**Electronic supplementary material:**

The online version of this article (doi:10.1186/s12974-014-0150-2) contains supplementary material, which is available to authorized users.

## Background

According to the last update reported by Lee and colleagues [1], the global incidence of traumatic spinal cord injuries (SCI) was estimated in 2007 at 23 cases per million worldwide. Reported SCI cases mainly concern young adult men, for the most part victims from motor vehicle accidents and falls [2]. Cervical and lumbar spines are the most commonly affected regions, inducing respectively tetraplegia and paraplegia. Patients suffer from motor impairments, spasticity, neuropathic pain, reflexive, sphincter, sexual and sensitive troubles, accompanied by highly disabling financial and social issues. Although experimental and clinical research have provided significant improvements in medical management and clinical recuperation after SCI in the last decade, no treatment allows complete functional recovery of patients, whatever the considered therapeutic strategy.

The development of such efficient treatments should first be based on the complete understanding of SCI physiopathological events. Those events are gathered in three major phases (acute, sub-acute and chronic), as previously reviewed [3,4]. Briefly, 1) the acute phase events after traumatic SCI and spinal shock encompass axonal disruption and neuronal death, blood supply default and ischemia, edema, invasion of granulocytes, disruption in ionic balance and neurotransmitter release; 2) the sub-acute (intermediate) stage starts around 7 days after the lesion and is characterized by further oxidative stress taking place by lipid peroxidation and free-radical production, as well as by the recruitment of macrophages and lymphocytes, which secrete cytokines and promote the development of an inflammatory environment; 3) the chronic phase arises after a few weeks to months, encompassing continuous alteration of ionic balance, apoptosis of oligodendrocytes and consequent demyelination, cavities and astroglial scar formation, persisting for years. Overall, those unfavorable events hamper axonal regrowth and functional recovery.

Accordingly, administration of high doses of methylprednisolone in the first hours after SCI was shown to reduce lesion extent and to limit motor decline in patients [5]. Up to now, corticosteroid administration remains the most efficient attempt to cure SCI patients by counteracting the inflammatory reaction. However, no complete regeneration can be achieved despite great advances, and thus, the fine-tuning of the inflammatory reaction should be more precisely considered.

**Figure 1:**
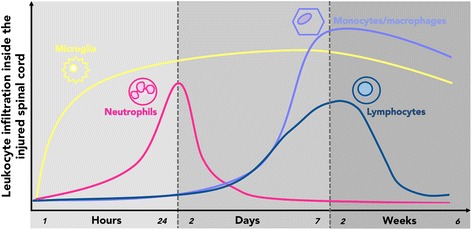
**Global temporal sequence of leukocyte recruitment of the spinal cord after injury in rodents.** (Adapted from [6]).

As already mentioned, host inflammatory response after SCI largely contributes to the elaboration of unfavorable tissue environment. Paradoxically, several studies pointed out that the inflammatory reaction could be mandatory in order to initiate efficient tissue repair [6]. Basically, early inflammatory events involve sequential recruitment of three main types of peripheral immune cells: 1) neutrophils are the first inflammatory cells to arrive at the site of injury, with a peak at 24 hours post-injury. Those cells phagocyte and clear debris, secrete proteases, elastase, myeloperoxidase and release reactive oxygen species (ROS); 2) circulating monocytes/macrophages are subsequently recruited (peak at 7 days post-injury), release cytokines such as TNF-α, IL-1β, nitric oxide, prostaglandins and leukotrienes, and also exert important phagocytic abilities; 3) lymphocytes progressively invade the lesion site, concomitantly to macrophages and secrete cytokines in the lesion epicenter. However, the number of recruited lymphocytes remains low compared to other cell types [7–9] (Figure 1).

Noteworthy, microglial cells of the spinal cord tissue contribute to the inflammatory reaction as well (reviewed in [10]), even if the distinction between those resident macrophages and peripheral blood-derived macrophages is still difficult to establish and their respective roles in SCI remain under investigation. Indeed, it appears that microgliocytes and peripheral monocytes differentially contribute to SCI recovery [11], and present distinct timeframes of action and phagocytic activities [12].

Macrophages have been the most studied immune cells in the context of spinal cord inflammation for many years and are now considered as crucial for tissue repair and functional recovery [13,14]. Exciting experimental results even led to clinical application of autologous macrophage-based therapies for SCI, even if further investigations are needed to characterize the significant effect of such interventions [15,16].

Nonetheless, scientists kept on delineating the mechanisms by which monocytes/macrophages were acting in the spinal cord after traumatic injuries. Two subtypes of macrophages were recently identified and classified as classically activated M1 macrophages, and alternatively activated M2 macrophages. Basically, M1 macrophages secrete IL-1β, TNFα and ROS, promoting tissue destruction and killing of parasites, whereas M2 macrophages secrete IL-10, IL-1RA or chemokines and induce tissue remodeling [17,18]. Both of those subtypes exert contrasting immunomodulatory actions in pathological conditions and especially in SCI [19,20]. Therefore, it appears that M2 macrophages are of great interest with regard to SCI therapy, as recently reviewed [21].

Altogether, existing evidence reveals that scientists reach a consensus about the required role of immunity and inflammation in nervous system disorders, and in spinal cord injuries in particular, while the understanding of molecular and cellular mechanisms by which each type of immune cells is acting is still under progress. However, as inflammation essentially implies sequential recruitment and activation of neutrophils and macrophages, it is surprising to note that the roles of the former are quite less known compared to the roles of the latter. Therefore, in this review, we will gather information about neutrophils and detail what is known about the different actions they could exert in the damaged nervous tissue, in an effort to reconsider the controversial role of those intriguing cells in spinal cord traumatic lesions.

## Neutrophils - origin, identification and general role in inflammatory response

**Figure 2:**
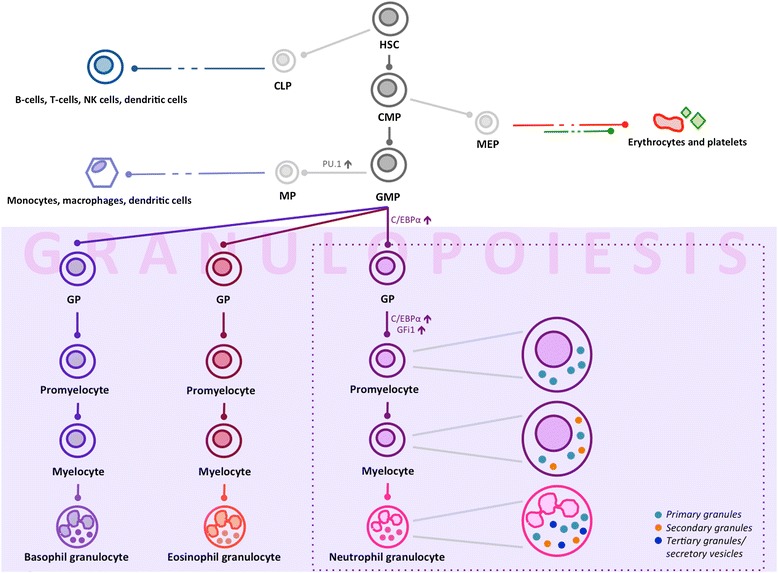
**Schematic view of granulopoiesis and neutrophil terminal differentiation.** CLP, common lymphoid progenitor; CMP, common myeloid progenitor; GMP, granulocyte/monocyte progenitor; GP, granulocyte progenitor); HSC, hematopoietic stem cell; MEP, megakaryocyte/erythroid progenitor; MP, monocyte/macrophage progenitor; NK, natural killer.

Granulocytes are a subset of white blood cells characterized by their polylobulated nucleus and by their cytoplasmic granules, which are differentially identified by cytological stainings and allow distinction between basophil, eosinophil and neutrophil granulocytes. Granulocytes arise from granulo-monocytic progenitors in the bone marrow (Figure 2). Primary cell fate determinants of the granulocytic and monocytic lineages are transcription factors PU.1 and C/EBPα. High levels of PU.1 promote a macrophage differentiation program through the secondary determinants Egr1,2/Nab-2, while repressing neutrophil-specific genes. Conversely, elevated levels of C/EBPα and secondary GFi-1 induce granulocytic differentiation, while antagonizing monocyte development (as reviewed by [22,23]). Primary granules appear at the promyelocyte stage and contain microbicidal proteins and acid hydrolases such as myeloperoxidase and lysozyme. Promyelocytes then develop into myelocytes, which display secondary or specific granules of neutrophilic, eosinophilic or basophilic cytochemical characteristics, and contain other hydrolases and chemotactic factors. Tertiary granules include secretory vesicles containing plasma proteins, and gelatinase granules. While primary granules are discharged exclusively into phagosomes, secondary and tertiary granules are released both outside the cell and in the extracellular medium.

A network of hematopoietic growth factors and cytokines (for example, granulocyte colony-stimulating factor (G-CSF)) regulates the production of granulocytic cells. G-CSF is a major regulator of neutrophilic granulocyte production and modulates the proliferation, survival, maturation and functional activation of these cells [24]. By activating the release of proteases from granulocytes, G-CSF induces a massive egress of immature cells from the bone marrow, including hematopoietic and non-hematopoietic stem cells, as well as granulocytic progenitors and precursors [25].

During bacterial infection or traumatic lesion, neutrophils are recruited from the bloodstream and migrate across endothelial barriers to reach the inflammatory site, being highly sensitive to chemoattractant signals such as IL-8, interferon-gamma and C5a. Other signals such as the CXCL12-CXCR4 or CXCL1/2-CXCR2 signalization pathways also regulate neutrophil mobilization and activation in inflammatory conditions, including in nervous system disorders [26–30]. Once recruited at their site of action, neutrophils roll and adhere to endothelial barriers before crossing over; they reach the lesion and secrete cytokines, release their cytoplasmic secondary and tertiary granule content, phagocyte cell debris, and form neutrophil extracellular traps [31], altogether clearing the lesioned tissue and/or microbes in a complex network of pathways. Cellular and molecular details concerning recruitment and activity of neutrophils in health and disease are reviewed in depth in [32,33].

Granulocytes are specifically identified by the expression of surface antigens CD66b and CD11b/c, which are responsible for adhesion and cell-cell interaction; and CD13, CD16 or CD88 (among others) mediating different aspects of the immune response. Murine granulocytes also express Ly6g and Ly6C members of the Ly6 family, potentially involved in neutrophil recruitment and migration [34]. These markers are often used for leukocyte subset identification and targeted in antibody-mediated depletion strategies [34,35], providing numerous insights into neutrophil biological function in a wide panel of domains.

## Neutrophil implication in spinal cord injuries

Neutrophils are usually considered as the “bad guys”, bluntly accumulating in the inflammatory core of a tissue lesion, secreting proteases, oxidative and tissue-degrading enzymes, thus elaborating a harmful tissue environment. Likewise, most of the studies describe them as detrimental actors. More specifically, neutrophils have been described to promote neurotoxicity on dorsal root ganglia neurons via the activity of matrix metalloproteinase 9, generation of ROS and secretion of TNF-α [36]. Cell-cell contact between neutrophils and neurons also seem to generate cytotoxicity [37].

Very few papers have focused on the role of neutrophils in SCI models, but their detrimental action was mainly highlighted as an effect/consequence of other treatments and conditions. Indeed, in most conditions, a lower neutrophil accumulation in the lesion was associated with reduced pro-inflammatory cytokines, reduced apoptosis and oxidative stress and significant motor recovery (see Additional file 1: Table S1). Besides underlining the deleterious effect of neutrophils, these studies also provided clues about the different ways in which these cells are recruited in the injured tissue. For instance, it was shown that neutrophil infiltration in the damaged spinal cord was reduced after blocking the leukotriene B4/BLT1 receptor signaling [38], after inhibiting phosphodiesterase 4 [39] or in absence of myeloperoxidase [40].

The role of the NF-κB signaling pathway was suggested, both in neutrophil invasion and in neutrophil activity in the lesion. Indeed, the blockade of inhibitor of NF-κB kinase subunit β (IKKβ) neutralized the secretion of CXCL1 and the subsequent neutrophil infiltration, but also the expression of pro-inflammatory genes, simultaneously improving tissue preservation and motor function [41].

Together with the chemokines CCL2 and CXCL2, CXCL1 was proposed as a neutrophil chemoattractant, which would be secreted by spinal cord astrocytes under IL-1 receptor (IL-1R)/MyD88 signalization [42], and which even seemed to mediate neuropathic pain [43]. Consistently, the concentration of CXCL1 in the serum of SCI patients is increased in the first week following injury, compared to healthy patients [44].

All these results essentially classify neutrophils as unfavorable actors in the inflammatory response, still it appears that their roles in injury/repair processes need to be more specifically addressed. Indeed, as clearly depicted in Additional file 1: Table S1, the specific activity of neutrophils in the spinal cord is largely unknown. There is now increasing evidence that neutrophils also exert at least indirect beneficial effects, probably by initiating inflammation-associated tissue repair, thus prompting us to re-evaluate and nuance the beneficial/harmful role of neutrophils in the injured spinal cord.

Recent specific antibody-based methods of Ly6G/Gr-1^+^ neutrophil depletion [34,45] revealed that the presence of neutrophils unexpectedly reduced the levels of ROS in a spinal cord lesion [46]. Surprisingly, Stirling and colleagues showed that the depletion of Ly6G/Gr-1^+^ neutrophils impaired the functional outcome in SCI mice, by preventing early vascular recruitment, rolling and adhesion to endothelia, spinal cord tissue infiltration, and exacerbating CXCL1, CCL2, G-CSF and CCL9 production inside the spinal cord as a compensatory attempt [47]. This study definitively demonstrated, for the first time, that neutrophils were required for appropriate inflammatory reaction and subsequent tissue repair after SCI. A few months later it was shown that secreted leukocyte protease inhibitor (SLPI) was required for SCI recovery. SLPI is secreted by neutrophils and astrocytes in the spinal cord tissue [48], which highlights a hypothetical mechanism underlying positive neutrophil action. On the other hand, neutrophils accumulate in the injured spinal cord of mice lacking tenascin-C, while axonal fibers penetrate easier through the spinal cord tissue [49], suggesting that neutrophils could contribute to the elaboration of a suitable environment for axonal regeneration.

## Neutrophils in other nervous system disorders

Neutrophils [50] and oxidative stress [51] are frequently associated with the pathogenesis of Alzheimer’s disease (AD). However, this aspect is quite controversial as several studies suggested that the number and function of circulating neutrophils were reduced in AD patients [52–54] as a consequence of the disease. New insights in the physiopathological processes of AD showed that neutrophils migrate towards amyloid plaques, maybe suggesting a potential interaction worthy of thorough characterization [55] in order to specify the role of neutrophils in AD pathogenesis.

It is also well accepted that neutrophils are a key player of the regulatory sequence of experimental autoimmune encephalomyelitis (EAE), essentially recruited and activated by inflammatory chemokines such as CXCL1, CXCL2 or CCL2 [56–58]. They are also involved in blood brain barrier disruption during the onset of experimental autoimmune encephalomyelitis in mice, probably because of an increased IL-1R-dependent transmigration ability [59].

Despite the demonstration of the numerous detrimental consequences associated with neutrophils, there is a noticeable gap of knowledge about how neutrophils are properly working in brain and spinal cord injuries. Noteworthy, recently published data now tend to reverse the trend and suggest that the role of neutrophils could be more balanced than it seems. Whereas the contribution of neutrophils to tissue repair and recovery from experimental stroke was highlighted several years ago (as reviewed in [60]), recently published results addressed the specific impact of neutrophils in inflammation-induced regeneration of the optic nerve and in peripheral nerve regeneration (Additional file 1: Table S1). Indeed, Yin and colleagues demonstrated that neutrophils are recruited during the first three days after zymosan-induced optic nerve inflammation, and secrete high levels of oncomodulin. Interestingly, macrophages that reach the lesion later on also secrete oncomodulin. Oncomodulin is a 12-kDa calcium-binding protein, which is secreted by neutrophils and macrophages, and was previously demonstrated to support neural regeneration in retinal ganglion cells in culture [61] and in the optic nerve [62]. However, macrophages alone are not sufficient to induce regeneration in the optic nerve when neutrophil recruitment is specifically prevented [63], suggesting an essential role for neutrophils and their specific oncomodulin secretion inside the optic nerve.

Neutrophils have been designated as responsible for hypersensitivity/neuropathic pain occurring after peripheral nerve injury [64,65]. Once again, however, several observations suggest a role for neutrophils in peripheral nerve regeneration. It has been shown that axonal regrowth after peripheral nerve injury was abolished in the absence of myeloid cells, which are specifically required to clear myelin debris and secrete neurotrophic factors such as neurotrophin-3,4,5 and brain-derived neurotrophic factor. Interestingly, it seemed that spinal cord axons needed myeloid cell support as well to properly regenerate in a peripheral nerve graft [66].

## Granulocyte colony-stimulating factor: its implication in experimental spinal cord injury, and clinical data

G-CSF is an important regulating factor of neutrophil development, recruitment and activity in physiological and pathological conditions. As stated below, G-CSF is largely described in SCI experimental models and in clinical trials because of its plenty of properties, both on the hematological and neurological points of view. Importantly, a hypothetic G-CSF-dependent role of neutrophils in SCI would be worth considering. Details about G-CSF activity in SCI are therefore of significant interest in order to further define the precise aspects of the inflammatory response after lesion.

Fundamentally, G-CSF is a 19,6-kDa glycoprotein [67] that binds on a specific G-CSF receptor at the surface of hematopoietic stem cells from the bone marrow, promoting their proliferation and differentiation into granulocytes, which are further released in the peripheral bloodstream. G-CSF also modulates mature neutrophil proliferation, activation and recruitment, therefore playing a pivotal role in the regulation of inflammatory responses. G-CSF induces mobilization of bone marrow stem cells into the peripheral blood through an indirect mechanism involving degradation of adhesion molecules by proteases released from activated neutrophils [68]. On the other hand, it has been shown that G-CSF receptor was also expressed by neurons of the central nervous system, and that G-CSF has neurotrophic actions via anti-apoptotic, anti-excitotoxic and pro-neurogenic abilities [69] as particularly addressed in models of cerebral ischemia [70,71], amyotrophic lateral sclerosis [72,73] or in the study of memory and cognitive functions [74]. Therefore, besides being extensively used in the clinic to counteract chemotherapy-associated neutropenia [75] and collect hematopoietic stem cells by apheresis [76], G-CSF was also proposed as a therapeutic option for the treatment of SCI. Indeed, Japanese researchers recently applied G-CSF as a clinical treatment for patients suffering from SCI. Modest but non-negligible motor and sensory improvements were observed, whereas no adverse effects were reported [77–79], suggesting a potential beneficial effect of G-CSF in the therapy of SCI.

Different studies have already evidenced the beneficial effects of G-CSF in experimental models of SCI [80,81], which seem to be mainly associated with the prevention of excitotoxicity and apoptotic neuronal death, through several possible mechanisms [82]. G-CSF upregulates chaperone proteins, such as nucleophosmin-1 in motoneurons after spinal cord hemisection [83], reduces myeloperoxidase activity and lipid peroxidation [84] and increases glial cell line-derived neurotrophic factor and vascular endothelial growth factor A expression in glial cells after spinal cord ischemia [85]. It also appears that G-CSF-associated neuroprotection, through its mobilization capacity, is equivalent to bone marrow mononuclear stem cell-induced neuroprotection [86]. Besides acting on neural cells, G-CSF also modulates inflammatory reaction and immune cell recruitment and activation in the injured spinal cord. Recent data showed that G-CSF induces alternative activation of microglial macrophages, thus promoting tissue repair [87]. Combined with stem cell factor administration, G-CSF increases the number of activated microglial cells and oligodendrocytes [88], whilst saving oligodendrocytes from SCI-induced cell death by reducing IL-1β and TNF-α and up-regulating the anti-apoptotic protein Bcl-xL [89]. Additional data are now required to elucidate a putative intermediate role of neutrophils in G-CSF-mediated effects on SCI.

## Conclusions

Although the prevalent view emerging from the current literature depicts neutrophils in SCI as cells with harmful actions and effects, recent data strongly suggest that their inflammatory function could oppositely provide valuable outcome for tissue repair. Indeed, it appears that neutrophils have always been classically considered as damaging despite the lack of knowledge on their precise mechanisms of actions after SCI. While neutrophils have their own activity by secreting enzymes or other molecules, their fine interactions with other immune cells (for example, macrophages) may accurately guide the inflammatory process as well. Neutrophils are important inducers of the inflammatory sequence, as observed in models of rheumatoid arthritis [90], antibody-mediated inflammation [91], or acute respiratory distress syndrome [92]. Indeed, neutrophils set the stage for macrophages which phagocyte debris and clean the lesioned tissue, in different inflammatory conditions such as bacterial infection [93,94] or physical exercise [95], among others. Besides, neutrophils also interact with T lymphocytes and natural killer cells, as recently reviewed [96]. It has also been demonstrated that neutrophils can promote wound repair by releasing angiogenic factors such as IL-8 or vascular endothelial growth factor, and then inducing neovascularization, which is crucial for inflammation-mediated tissue remodeling [97,98].

In addition, neutrophil-induced positive effects could also be linked to intrinsic properties of sub-populations. As described in several types of cancers, tumor-associated neutrophils are classified according to their pro- or antitumoral global actions as N1 (pro-tumor) and N2 (anti-tumor) [99]. As no phenotypic analysis of neutrophils has ever been carried out in traumatic lesions of the nervous system, one can imagine that the same duality could be translated, just as it is the case for the M1/M2 macrophage dyad (see above).

Overall, the lack of knowledge about the proper role of neutrophils in SCI and repair now becomes blindingly obvious. Neutrophils have usually been considered as deleterious actors to target for reducing lesion extent; however, it is now clear that their role must be thoroughly questioned. Further information about their cellular and molecular mechanisms of action should provide new insights in the field of SCI, and also in neurological diseases in general.

## Additional file

Additional file 1: Table S1. Recruitment and activity of neutrophils in spinal cord injuries and other nervous system lesions.

## Abbreviations

AD: Alzheimer’s disease
G-CSF: granulocyte colony-stimulating factor
IKKβ: inhibitor of NF-κB kinase subunit β
IL: interleukin
IL-1R: interleukin-1 receptor
NF: nuclear factor
ROS: reactive oxygen species
SCI: spinal cord injuries
SLPI: secreted leukocyte protease inhibitor
TNF: tumor necrosis factor
