# Supplementary material for: Neutrophil contribution to spinal cord injury and repair
Source: J Neuroinflammation. 2014 Aug 28;11:150. doi: 10.1186/s12974-014-0150-2 (PMC4174328; doi:10.1186/s12974-014-0150-2)
Supplement: Additional file 1: Table S1. — Recruitment and activity of neutrophils in spinal cord injuries and other nervous system lesions. [file 12974_2014_150_MOESM1_ESM.docx]

|  | **Model** | **Recruitment of neutrophils inside the spinal cord/tissue** | | | | **Neutrophils associated with…** | **Ref** |
| --- | --- | --- | --- | --- | --- | --- | --- |
|  |  | ***Pathway*** | ***Pathway inhibition*** | ***Intermediate*** | ***Effects of inhibition*** |  |  |
| 1 | SC Comp | PDE4 | IC486051  (0,5 or 1 mg/kg)  6 days post injury | nd | 🡮 MPO activity  🡮 neutrophil infiltration  🡮 macrophage infiltration  🡮 lipid peroxidation  🡮 ROS formation  🡭 motor function | **🡭 APOPTOSIS**  **🡭 OXIDATIVE STRESS**  **🡮 MOTOR RECOVERY** | 39 |
| 2 | SC Cont | LTB4/BLT1 | ONO-4057 (10mg/kg)  5 days post injury  BLT1-KO mice | 🡮 CXCL1 ?  🡮 CXCL2 ?  🡮 CCL2 ? | 🡮 neutrophil infiltration  🡮 apoptotic cell number  🡭 motor function  🡭 axonal conduction  🡮 IL6  🡮 IL1β  🡮 TNFα  🡮 CXCL1  🡮 CXCL2  🡮 CCL2 | **🡭 APOPTOSIS**  **🡭 PRO-INF CYTOKINES**  **🡮 AXONAL CONDUCTION**  **🡮 MOTOR RECOVERY** | 38 |
| 3 | SC Cont | MPO | MPO-KO mice | nd | 🡮 HOCl  🡮 neutrophil infiltration  🡮 apoptotic cell number  🡭 tissue sparing  🡭 motor function | **🡭 APOPTOSIS**  **🡭 OXIDATIVE STRESS**  **🡮 MOTOR RECOVERY**  **🡮 TISSUE SPARING** | 40 |
| 4 | SC Hemisect | IKKβ | IKKβ-KO mice | 🡮 CXCL1  🡮 CCL2  🡮 CCL3  🡮 CCL4 | 🡮 neutrophil infiltration  🡮 macrophage infiltration  🡭 tissue sparing  🡭 motor function  🡮 apoptotic cell number  🡮 ROS formation | **🡭 APOPTOSIS**  **🡭 OXIDATIVE STRESS**  **🡮 MOTOR RECOVERY**  **🡮 TISSUE SPARING** | 41 |
| 5 | SC Hemisect | Tenascin C | Tenascin C-KO mice | nd | 🡮 fibronectin expression  🡭 neutrophil infiltration  🡭 lymphocyte infiltration  🡭 neurofilament expression | **🡮 FIBRONECTIN**  **🡭 AXONAL REGROWTH** | 49 |
| 6 | SC Cont | MyD88/IL1R | MyD88-KO mice IL1R-KO mice | 🡮 CCL2, CXCL1, CXCL2 by astrocytes | 🡮 neutrophil infiltration  🡮 macrophage infiltration | nd | 42 |
|  | **Model** | **Activity of neutrophils inside the spinal cord/tissue** | | | | **Neutrophils associated with…** | **Ref** |
|  |  | ***Pathway*** | ***Pathway inhibition*** | ***Intermediate*** | ***Effects of inhibition*** |  |  |
| 7 | SC Cont | SLPI | nd | nd | nd | **🡭 MOTOR RECOVERY**  **🡭 TISSUE SPARING** | 48 |
| 8 | ON lesion | Oncomodulin | Ly6G blocking  P1 (Ocm antagonist) | nd | 🡮 axonal regeneration | **🡭 AXONAL REGROWTH** | 63 |
| 9 | PN lesion | Phagocytosis  NT-3, NT4/5, BDNF | CD11b blocking | nd | 🡮 axonal regeneration  🡮 clearance of myelin debris  🡮 NT-3, NT4/5, BDNF secretion  🡮 angiogenesis  🡮 regeneration of SCI-axons in PN grafts | **🡭 AXONAL REGROWTH**  **🡭 PHAGOCYTOSIS**  **🡭 NEUROTROPHINS**  **🡭 ANGIOGENESIS** | 66 |

**Table 1**
